# Consilience of methods for phylogenetic analysis of variance

**DOI:** 10.1111/evo.14512

**Published:** 2022-05-19

**Authors:** Dean C. Adams, Michael L. Collyer

**Affiliations:** ^1^ Department of Ecology, Evolution, and Organismal Biology Iowa State University Ames Iowa USA; ^2^ Department of Science Chatham University Pittsburgh Pennsylvania USA

**Keywords:** Brownian motion, macroevolution, phylogenetic comparative methods, simulation/methods

## Abstract

Simulation‐based and permutation‐based inferential methods are commonplace in phylogenetic comparative methods, especially as evolutionary data have become more complex and parametric methods more limited for their analysis. Both approaches simulate many random outcomes from a null model to empirically generate sampling distributions of statistics. Although simulation‐based and permutation‐based methods seem commensurate in purpose, results from analysis of variance (ANOVA) based on the distributions of random *F*‐statistics produced by these methods can be quite different in practice. Differences could be from either the null‐model process that generates variation across many simulations or random permutations of the data, or different estimation methods for linear model coefficients and statistics. Unfortunately, because the null‐model process and coefficient estimation are intrinsically linked in phylogenetic ANOVA methods, the precise reason for methodological differences has not been fully considered. Here we show that the null‐model processes of phylogenetic simulation and randomizing residuals in a permutation procedure are indeed commensurate, and that both also produce results consistent with parametric ANOVA, for cases where parametric ANOVA is possible. We also provide results that caution against using ordinary least‐squares estimation along with phylogenetic simulation; a typical phylogenetic ANOVA implementation.

Biology in the 21st century is firmly entrenched in the big data revolution. The technological advances of recent years have enabled biologists to rapidly characterize thousands of genomic (Qin et al. 2015; Papageorgiou et al. 2018), phylogenomic (Young and Gillung 2020), morphological (Goswami et al. 2019), physiological (Orphanidou 2019), climatic (Stockwell 2006), and behavioral attributes (Kabra et al. 2013), from hundreds to thousands of observations representing individuals, populations, species, and communities. These large biological datasets, interrogated with mechanistic and phenomenological models (Otto and Day 2007; Maruvka et al. 2013; Connolly et al. 2017; Mitov et al. 2019; Otto and Rosales 2020), have extended the scope of inquiry in ecology and evolution, leading to major insights. Although computation‐intensive analysis in not new (Crowley 1992), significant challenges still remain in how to extract biological signal from large (Li and Chen 2014)—and even traditionally sized—datasets. In particular, sparse data tables, ill‐conditioned covariance matrices, convergence issues with likelihood estimation, and models that lack a known probability density distribution, all lead to computational and statistical complexities for evaluating trends in biological data.

Monte Carlo (simulation) and resampling methods (Manly 2007) have a rich history in evolutionary biology research (Martins and Garland 1991; Crowley 1992; Garland et al. 1993, 2005). As computers have become more powerful, biologists are increasingly turning to computation‐intensive approaches, especially for analyses that lack parametric solutions or for data that violate parametric assumptions. Computation‐intensive approaches can include simulation methods (Garland et al. 2005; Rangel et al. 2018; Cornell et al. 2019) and resampling procedures (Manly 2007; Collyer and Adams 2018). With respect to the former, a family of procedures known as *simulation‐based inference* methods are gaining prominence (Diggle and Gratton 1984; Gourieroux and Monfort 1993; Cranmer et al. 2020; Brehmer et al. 2020). These approaches, which include approximate Bayesian computation, likelihood and probability estimation, neural networks and neural learning (among other approaches), are particularly useful for characterizing models that describe complex biological dynamics, even when the probability distribution of the model is intractable (Beaumont 2019; Cranmer et al. 2020; Brehmer et al. 2020). Likewise, resampling procedures are increasingly used, especially for highly multivariate data that can preclude multivariate (*M*) analysis of variance (ANOVA). Resampling procedures are frequently used for multivariate statistics in MANOVA (Clavel and Morlon 2020), ANOVA based on dissimilarity matrices (Anderson 2001), or ANOVA using univariate‐like *F*‐statistics calculated from traces of covariance matrices (Collyer et al. 2015). These methods use random permutations of data or linear model residuals. Bootstrap resampling can also be performed for ANOVA (see, e.g., Figueiredo 2017), if resampling data with replacement is preferred.

Conceptually, simulation‐based and resampling‐based inference procedures, when used for hypothesis testing, are straightforward. First, random samples of observations are drawn (simulated) from a specified generating process that describes the biological null model under investigation. Next, test statistics are obtained for each simulated dataset, which compose null sampling distributions for the statistics. The chief difference is whether data are newly simulated, perhaps drawing a sample from, for example, a normal distribution, or redistributed in random permutations of the existing data or linear model residuals. Performed many times, either approach allows a sampling distribution proxy (of a real but perhaps intractable sampling distribution) of a test statistic to be empirically generated, and inferences about the observed data are made based on the location of the observed statistic in the sampling distribution. It has been shown that empirical sampling distributions obtained from simulation‐based inference approaches can accurately approximate both likelihood profiles (Diggle and Gratton 1984; Gourieroux and Monfort 1993) and theoretical sampling distributions of summary statistics (Kac 1949; O'Hara 2019) for models that could equally use parametric probability distributions as proxies for sampling distributions. Likewise, various tests based on resampling data or residuals of linear models have been shown to have good statistical properties in terms of type I error, statistical power, and asymptotic convergence on exact tests (Anderson and Robinson 2001; Manly 2007).

One challenge for comparative data is that the observations under scrutiny (e.g., species) are correlated with one another due to shared phylogenetic history (Felsenstein 1985). Failure to account for phylogenetic history in analysis of data can result in spurious conclusions (Garland et al. 2005; Rezende and Diniz‐Filho 2011). The nonindependence of observations because of phylogenetic relatedness can be described by an object covariance matrix, C, which describes the expected correlation among species due to common ancestry from a Brownian motion (BM) model of evolutionary divergence (Grafen 1989; Martins and Hansen 1997; Rohlf 2001; O'Meara et al. 2006). This matrix has had multiple uses in phylogenetic comparative methods (PCMs). It has been used to simulate data for species with expected phylogenetic relatedness as a null‐model process for distributions of *F*‐statistics in ANOVA and analysis of covariance (Garland et al. 1993). For such analyses, many thousands of datasets are generated and one or more *F*‐statistics are calculated for linear model effects, using ordinary least squares (OLS) estimation of linear model coefficients. A distribution of *F*‐statistics from the random datasets is used as reference to calculate the percentile of the observed *F*‐statistic (from the real data) as a *P*‐value for a hypothesis test. This simulation‐based method assures that the null‐model process accounts for phylogenetic correlations in the data. C can also be used to condition the estimation of linear model coefficients, such that residuals are independent, via generalized least squares (GLS; Martins and Hansen 1997). Letting Ω=f(C), a transformation of the C matrix, ANOVA can be performed on linear model effects, with the violation of the assumption of independent observations comfortably abated by GLS estimation. This approach can yield the same linear model coefficients as using phylogenetically independent contrasts (PIC) (Felsenstein 1985) for single‐factor linear models and if Ω=C. Therefore, parametric ANOVA results for the effects based on PIC or GLS coefficients will be the same if C is not transformed in any way (Blomberg et al. 2012).

Using simulated datasets is a nonparametric PCM for testing linear model effects—henceforth, abbreviated here as sim‐pANOVA—and phylogenetic (P) GLS estimation is a PCM that allows for parametric ANOVA under certain circumstances. However, there are cases (e.g., multivariate data) that might be better approached with a nonparametric method. Several permutation tests using PGLS or PICs have been recently introduced. Klingenberg and Marugán‐Lobón (2013) introduced a method of obtaining PICs, variable by variable, which can be concatenated in a matrix whose rows can be shuffled in a permutation procedure. Adams and Collyer (2015) demonstrated this method had inferior type I error rates, compared to randomizing residuals in a permutation procedure (RRPP) (Adams 2014; Collyer et al. 2015) and performing ANOVA by calculating univariate‐like *F*‐statistics based on PGLS coefficients in random permutations to generate distributions of *F*‐statistics, much like the purpose of sim‐pANOVA. The RRPP‐ANOVA method was refined by Adams and Collyer (2018b) to use phylogenetically transformed residuals, which had better and appropriate type I error rates under more conditions. In comparison to sim‐pANOVA, RRPP‐ANOVA had greater statistical power, although both methods had appropriate type I error rates (see also, Revell 2013). Furthermore, for circumstances that parametric ANOVA following PGLS was appropriate (assumptions met), the random *F* distributions tracked the parametric *F*‐distribution, making the methods commensurate (Adams and Collyer 2018b).

The need for nonparametric PCMs apply inasmuch as the need to analyze complex data exists, but under conditions that traditional, parametric ANOVA is possible, one would expect consistent results between parametric and simulation‐based or permutation‐based (resampling) methods. However, previous comparison of sim‐pANOVA and RRPP‐ANOVA did not take into account that both methods have two components—estimation and a null‐model process—that could be considered independently to ascertain why one method performs better than another with regard to type I error rate or statistical power. Estimation simply refers to whether OLS or GLS is used to estimate coefficients, which thus impacts the calculation of *F*‐statistics in ANOVA. A null‐model process is the process that generates random outcomes over many permutations from a null model. Simulation of residuals or RRPP might be sufficient null‐model processes, provided the fixed effects of a null model are preserved (Collyer et al. 2015). Alternatively, simulating or randomizing data (rather than residuals) for hypothesis tests on multiple effects of a linear model would be less appropriate, as such a strategy would lack approximate exchangeability (Commenges 2003); the data would not have the same expectation (mean) as the error (zero). Recent statistical research for RRPP‐ANOVA has demonstrated that phylogenetically transformed residuals from null models that use GLS estimation of coefficients have appropriate exchangeability (sensu Commenges 2003), meaning that random pseudo‐data created by this resampling procedure have approximately the same null‐model residual variance for single traits (covariances and variances for multiple traits), across permutations. The simulation of data using the same C matrix in simulation runs should also produce datasets that have similar variance. We are, however, unaware of any previous research that has directly compared the consistency of simulation and permutation of transformed residuals as null‐model processes.

As described, sim‐pANOVA combines simulation of data (from a BM model of evolutionary divergence) as a null‐model process, with estimation by OLS. By contrast, RRPP‐ANOVA combines resampling (randomization) of transformed residuals as a null‐model process, with estimation by GLS. (The typical application of sim‐pANOVA is to simulate data, rather than residuals, but for a single‐factor model, this is not an issue as the null model contains only an intercept to estimate the mean.) It might be clear that OLS versus GLS estimation is one instance where greater statistical power should be expected with PGLS, as in RRPP‐ANOVA. However, while GLS generally exhibits higher statistical power when compared with methods that ignore the correlations among observations (Revell 2010), if Ω is not estimated properly, parameter estimates can be biased, and model evaluation procedures can be compromised (Gourieroux et al. 1984; Zeger et al. 1988; Koreisha and Fang 2001; Chavance and Escolano 2016; for discussion in a phylogenetic context, see Revell 2010; Blomberg et al. 2012).

It is possible to separately evaluate estimation and the null‐model processes used in sim‐pANOVA and RRPP‐ANOVA. In this study, we use a 3×3×2 design of data type (three different levels of phylogenetic signal), *F*‐statistic calculation (OLS or two different forms of GLS—see below), and null‐model process (simulation or RRPP), to better ascertain whether it is the null‐model process or estimation, or both, that leads to differences in performance (statistical power and other attributes) among methods. We compare empirically generated sampling distributions of *F*‐statistics to parametric *F*‐distributions, and we evaluate the consistency of *F*‐statistics, *P*‐values, and effect sizes estimated from the various methods. Finally, we discuss under which conditions a particular methodological approach would be best.

## Methods

Throughout our methods and results, we distinguish between “data generating models” and “analytics.” The former focuses on how data were simulated with known properties (such as with evolutionary correlations) and the latter refers to how data were analyzed, as if obtained without knowledge of the properties that were simulated. Analytics refers to the choice a researcher might make with data, such as choosing between simulation‐based or permutation‐based approaches, and between OLS and GLS solutions for estimating coefficients, and thus, ANOVA statistics.

*Simulation strategy*. To discern how the choice of analytics affect the approximation of empirical sampling distributions and statistical power, we performed a series of stochastic sampling experiments. The sampling experiments were based on a linear model, using a single‐factor ANOVA design. We simulated data with an expected variance, $\Sigma =\Omega \sigma^{2}$, with $\sigma^{2}=1$ (standard normal distribution) and Ω varied for three distinct data‐generating models: phylogenetic independence, phylogenetic correlation based on a BM model of evolutionary divergence, and an intermediate amount of phylogenetic correlation; i.e., three levels of phylogenetic signal in the data. For any simulated dataset, three different coefficient estimation methods were used. Coefficients were estimated as, $\hat{\beta }={\left(X^{T}{\hat{\Omega }}^{-1}X\right)}^{-1}X^{T}{\hat{\Omega }}^{-1}y$, with three different versions of $\hat{\Omega }$, corresponding to OLS, GLS based on a $\hat{\Omega }=C$ (the covariance matrix representing a BM model of evolutionary divergence), and GLS using a $\hat{\Omega }$ matrix, based on a maximum‐likelihood fit of data to the phylogenetic tree, relative to phylogenetic signal. Two distinct null‐model processes were also used to generate empirical sampling distributions of *F*‐statistics: simulation of residuals using $\hat{\Sigma }=C{\hat{\sigma }}^{2}$, where ${\hat{\sigma }}^{2}$ was calculated following coefficient estimation, and RRPP. This 3×3×2 design made it possible to isolate the impact of estimation and null‐model process on ANOVA results, whether data were independent with respect to phylogeny.

For any simulation run, we generated 100 or 200 “observed” datasets, each containing n=250 independent observations drawn from a generating model. The datasets corresponded to 100 or 200 pure‐birth phylogenies containing n=250 species each. Datasets (*y*) were simulated as $y=X\beta +\varepsilon_{N(0,\Sigma )}$, where ε was a vector of independent residuals drawn from a normal distribution, N(0,Σ); the model design matrix, X, was a matrix comprising 0s and 1s as dummy variables to indicate group association (with no a priori association to *y*) for 10 groups, each with 25 species; and β was a vector of coefficients to add group effects (difference in means between groups). (Initial trials that varied group number and the number of species per group indicated that these variables were not consequential for the results, but using 10 groups produced sampling distributions that were easy to compare among methods.) For these simulations, no group effect could be included (i.e., all β=0), which was tantamount to generating random response data ($y=\varepsilon_{N(0,\Sigma )}$) and randomly assigning those observations to groups.

*Comparison of sampling distributions*. For our first set of simulations, we varied Ω, only, and set β=0; i.e., no expected differences among groups. One version of Ω was an unscaled matrix based on a BM model of evolutionary divergence (Felsenstein 1985; Grafen 1989; Rohlf 2001; Huey et al. 2019), that is, Ω=C. The other two changed the amount of covariance (phylogenetic signal in the data) by the scaling parameter, Pagel's λ, which scales the internal branch lengths of the tree, optimizing the fit of the data to the tree (Pagel 1997). Letting D=diag(C), a diagonal matrix of only the phylogenetic variances of C, a rescaled form of C is Ω=λ(C−D)+D (Collyer et al. 2022). We used λ=0,0.5, and 1 (sensu Clavel and Morlon 2020) to scale covariance matrices from random trees, yielding datasets with phylogenetic independence, intermediate phylogenetic signal (correlation), and phylogenetic signal as expected with BM (unscaled tree), respectively. These simulations allowed us to consider the correspondence between null sampling distributions using different combinations of estimation and null‐model process, in the absence of group effects.

Because data were simulated such that assumptions for parametric ANOVA should be met, we compared results to parametric ANOVA in several ways. First, we mapped empirical *F*‐distributions on a parametric *F*‐distribution. Doing so revealed how well the combinations of null‐model process and coefficient estimation matched theoretical expectation. Second, we estimated *P*‐values as the percentiles of observed cases in their corresponding distributions, which could be compared to *P*‐values estimated via integration of the probability density function of the *F*‐distribution, based on degrees of freedom. Third, we estimated effect sizes as *Z*‐scores, the standard deviate of observed *F*‐statistics in their normalized distributions (*sensu*, Adams and Collyer 2018b). Finally, “pairs” plots of *P*‐values and *Z*‐scores were used to evaluate the consistency of statistics among the different methods, including parametric ANOVA for *P*‐values.

*Comparison of statistical power*. For our second set of simulations, we repeated the design of the first set of simulations, but varied the first β parameter, from 0 to 8, in increments of 2. (This approach increased the mean of the first group from the other groups by 0, 2, 4, 6, and 8, to create an effect.) These simulations allowed us to consider differences in type I error rate (β=0) and statistical power (β>0) among different combinations of estimation and null‐model process. Initial trials suggested that 200 simulated trees were sufficient to obtain a reliable estimate of null hypothesis rejection rate at an expected significance level of α=0.05.

In all simulation runs, coefficient estimation was performed on simulated data in three different ways. OLS estimation—as was used by Garland et al. (1993)—does not attempt to account for phylogenetic correlation in the data, even though data might be simulated to have phylogenetic correlations; that is, $\hat{\beta }={\left(X^{T}X\right)}^{-1}X^{T}y$, where ^*T*^ represents vector transposition, −1 represents matrix inversion, and y is a vector of data. GLS estimation uses Ω in estimation of coefficients; that is, $\hat{\beta }={\left(X^{T}{\hat{\Omega }}^{-1}X\right)}^{-1}X^{T}{\hat{\Omega }}^{-1}y$. $\hat{\Omega }$ for coefficient estimation can be determined in one of two different ways. First, it can be assumed to correspond to either a BM model of evolutionary divergence or the covariances can be scaled by an a priori notion of what the covariance should be. For example, scaling the covariances by λ=0 produces coefficients that are no different from OLS estimation; that is, OLS estimation is the same as assuming phylogenetic independence in GLS estimation. Second, a maximum‐likelihood estimate of Ω can be obtained by finding the value of λ that maximizes the likelihood of the data, given the tree; that is, $\hat{\Omega }=\Omega \left(\hat{\lambda }\right)$ (see Collyer et al. 2022). We performed coefficient estimation for λ=0 (OLS), λ=1 (typical with PGLS analysis), and the maximum‐likelihood estimate of λ, $\hat{\lambda }$ for every dataset produced in every simulation run.

*F*‐statistics were calculated for every model, from the coefficients estimated, as $F=\left(k-1\right){\left(n-k\right)}^{-1}{\left(r-r_{0}\right)}^{T}{\hat{\Omega }}^{-1}\left(r-r_{0}\right){\left(r^{T}{\hat{\Omega }}^{-1}r\right)}^{-1}$, where r is a vector of residuals found as $r=y-X\hat{\beta }$ and *k* is the number of model parameters. The residuals, $r_{0}$, were likewise calculated from a model with only an intercept, $X_{0}$ and its estimated coefficient, ${\hat{\beta }}_{0}$. The two null‐model processes were applied to each case, using 999 random permutations (which along with observed statistics generated distributions of 1000 random *F*‐statistics). For simulation as a null‐model process, $\varepsilon_{N(0,\Sigma )}$ were newly obtained; for RRPP, $\varepsilon_{N(0,\Sigma )}$ were estimated by randomizing the transformed residuals of a null model containing only an intercept (mean). One important caveat is that only if new data are simulated are the null‐model process and coefficient estimation truly independent, but resampling residuals means they are intrinsically linked.

This can be appreciated by the formula for residual variance for univariate data and a model with *k* parameters, ${\hat{\sigma }}^{2}={\left(n-k\right)}^{-1}{\left(y-X\hat{\beta }\right)}^{T}{\hat{\Omega }}^{-1}\left(y-X\hat{\beta }\right)$, which can be equivalently written with Cholesky decomposition of $\hat{\Omega }$ as, ${\hat{\sigma }}^{2}={\left(n-k\right)}^{-1}{\left(y-X\hat{\beta }\right)}^{T}{\left(\Psi \Psi^{T}\right)}^{-1}\left(y-X\hat{\beta }\right)$. Thus, the equation can be updated as ${\hat{\sigma }}^{2}={\left(n-k\right)}^{-1}{\left(\Psi^{-1}\left(y-X\hat{\beta }\right)\right)}^{T}\left(\Psi^{-1}\left(y-X\hat{\beta }\right)\right)$, where $\left(\Psi^{-1}\left(y-X\hat{\beta }\right)\right)$ are the phylogenetically transformed residuals and exchangeable units under the null hypothesis (Adams and Collyer 2018b). Because transformed residuals require Ω, both in the calculation of $\hat{\beta }$ and the transformation of the residuals, the null‐model process (randomization of residuals) is not independent of coefficient estimation.

In comparing the different combinations of null‐model process and estimation, the answers for the following four questions were sought. Do the distributions of random *F*‐statistics comport as expected, compared to theoretical (parametric) distributions? From the distributions of random *F*‐statistics, do the different combinations produce consistent results with regard to null hypothesis tests (correlation of *P*‐values across simulation runs)? From the distributions of random *F*‐statistics, do the different combinations produce consistent effect sizes (correlation of *Z*‐scores across simulation runs)? Do the different combinations have similar statistical power over a range of simulated effects? All simulations were performed in R 4.1.2. (R Core Team 2021). The functions, phytools::pbtree (Revell 2012) and geiger::rescale.phylo (Harmon et al. 2008), were used to randomly generate and rescale phylogenetic trees, respectively. Support functions from RRPP (Collyer and Adams 2018, 2021b) and geomorph (Adams et al. 2021; Baken et al. 2021) were used along with new functions written by the authors for simulations and analysis. All R scripts used are provided as supporting information.

## Results

**Figure 1:**
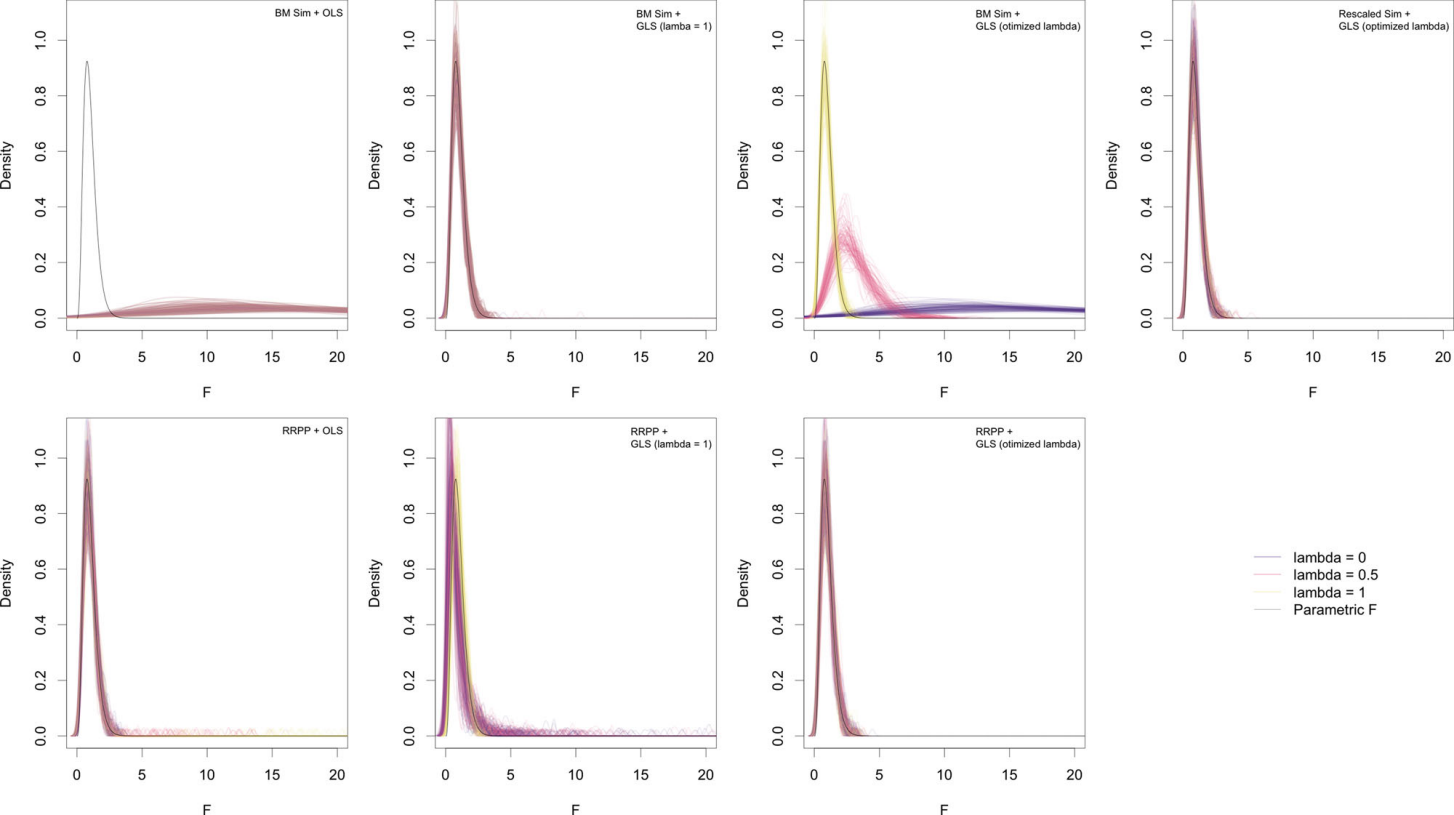
Density plots for random *F*‐statistics, from different combinations of estimation and null‐model process, illustrating sampling distribution behavior. One hundred density curves for every level of simulated λ (colored differently) are overlayed in every frame. Parametric *F*‐distributions are shown as black curves.

*Comparison of sampling distributions*. Our results indicated that when the null‐model process and estimation were commensurate—estimation matched the type of Ω matrix used to generate random outcomes—random *F*‐statistics formed distributions that were consistent with theoretical expectation, irrespective of whether the null‐model process used simulation or RRPP (Fig. 1). Most notably, when GLS was performed with optimization of λ, consistent empirical *F*‐distributions were produced, irrespective of null‐model process, and matched well to the parametric *F*‐distribution. Other cases in which there was a good match appeared to be incidental. For example, simulating data with a BM process and using GLS estimation with λ=1 produced consistent distributions, regardless of whether data were simulated with λ=0,0.5, or 1. The most notable departures from this trend occurred with simulation with a BM process as a null‐model process, but OLS estimation, or GLS estimation with optimized λ, when the data were obtained from a model other than λ=1 (Fig. 1). There were also noticeable inconsistencies among distributions when RRPP was performed with λ=1, for data not simulated from a model with λ=1 (BM). However, the mismatches between these distributions and the parametric *F*‐distribution were far less severe than those between optimized GLS estimation for simulation of BM data as null‐model process.

Most notably, the combination of BM simulation as a null‐model process and OLS estimation—the combination used in sim‐pANOVA—did not produce empirical *F*‐distributions that resemble parametric *F*‐distributions. These results were basically replicated with optimized λ in GLS estimation, presumably because the optimized value would be near or equal to 0. Collectively, the results indicate that (1) λ should be optimized and (2) if this is done, and if simulation is used, $\hat{\Omega }$ should be a rescaled form of C (the internal branches of the tree are rescaled) for the null‐model process. This combination of simulation and GLS estimation using a rescaled C matrix and RRPP using the rescaled C matrix based on $\hat{\lambda }$ yielded unequivocally consistent distributions, regardless of the value of λ.

**Figure 2:**
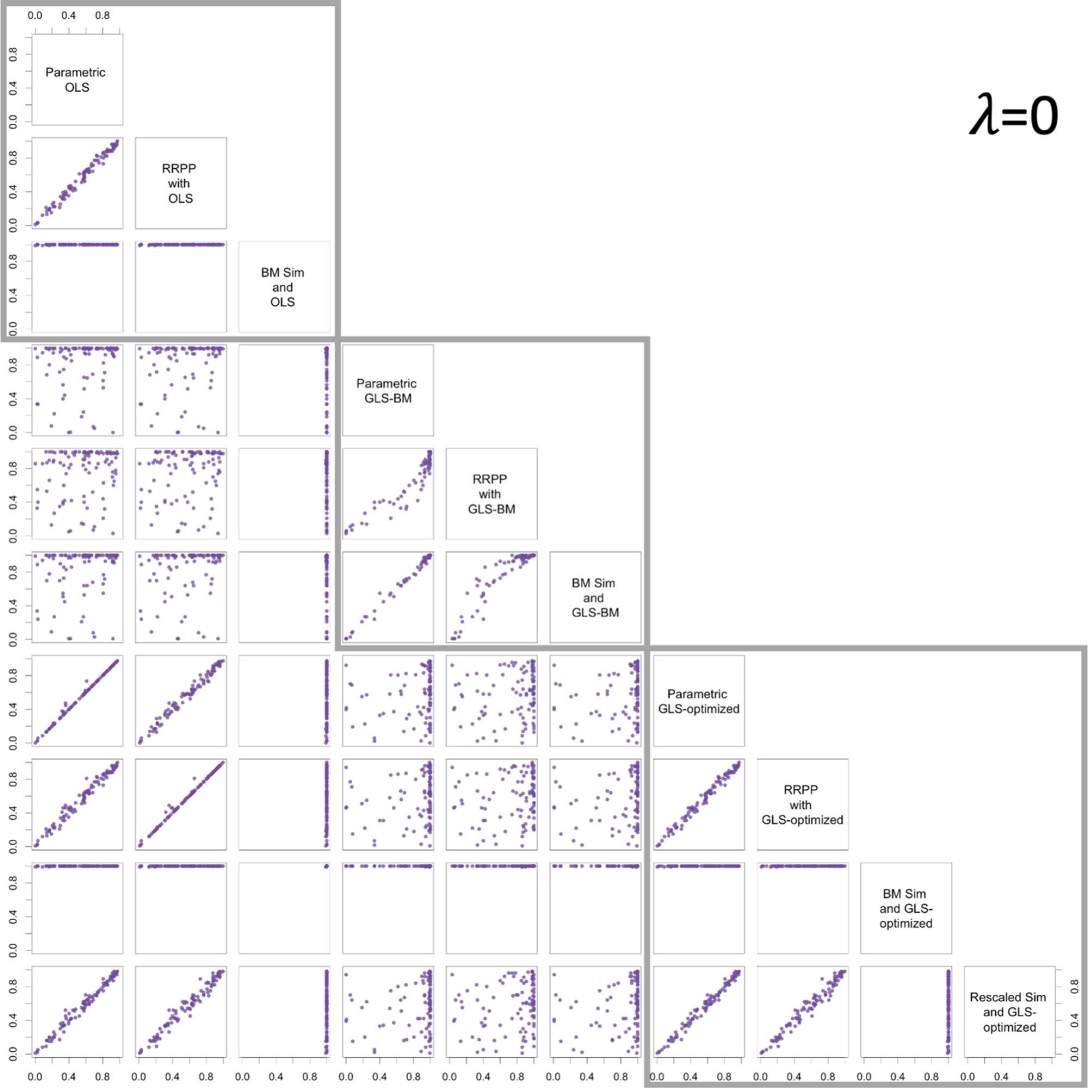
Pair plots for *P*‐values from different combinations of estimation and null‐model process, for data generated with λ=0, illustrating the consistency of different combinations of null‐model process and estimation. Gray boxes surround plots with the same estimation method.

**Figure 3:**
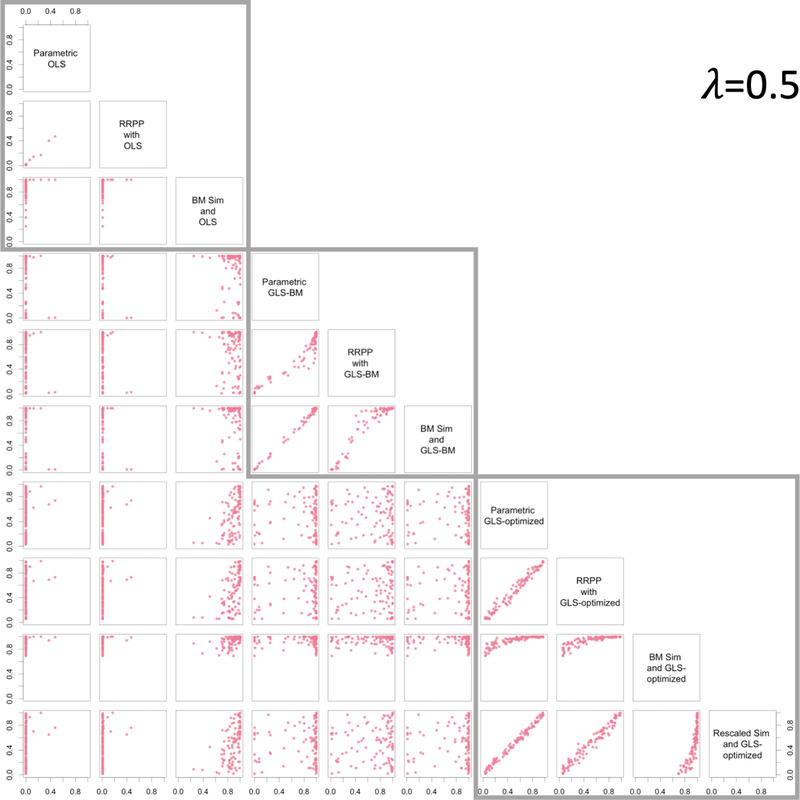
Pair plots for *P*‐values from different combinations of estimation and null‐model process, for data generated with λ=0.5, illustrating the consistency of different combinations of null‐model process and estimation. Gray boxes surround plots with the same estimation method.

**Figure 4:**
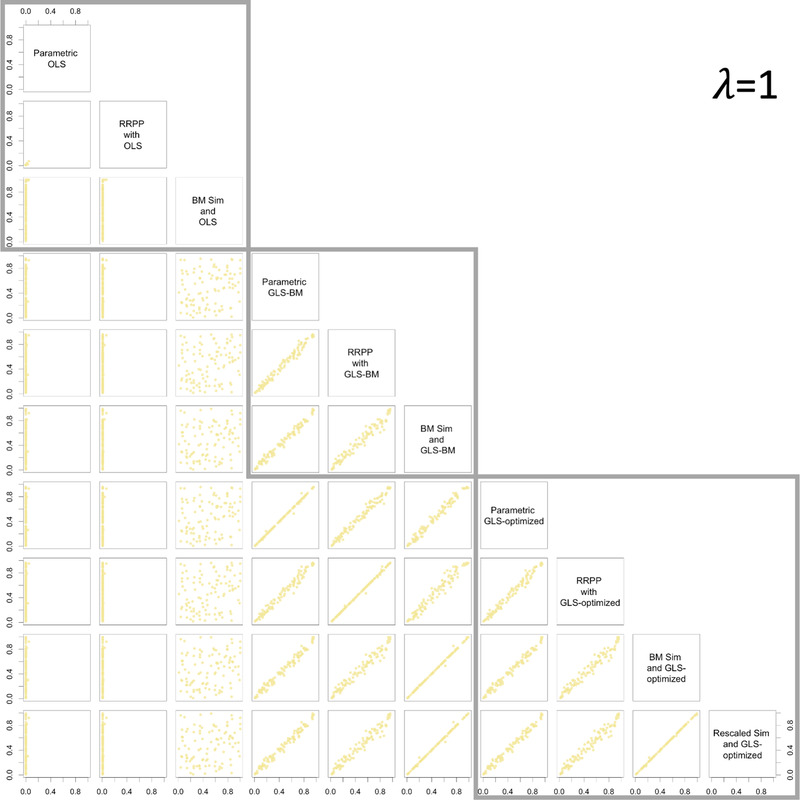
Pair plots for *P*‐values from different combinations of estimation and null‐model process, for data generated with λ=1, illustrating the consistency of different combinations of null‐model process and estimation. Gray boxes surround plots with the same estimation method.

The consistency of empirical sampling distributions with parametric distributions might not be a cause for concern for hypothesis tests (more on this below), provided there is consistency in null hypothesis test outcomes. In general, the correspondence between *P*‐values was noteworthy (Pearson *r* > 0.99) for comparable methods whether using OLS or either form of GLS, and for data generated with no phylogenetic signal (Fig. 2), data with intermediate phylogenetic signal (Fig. 3), or data with phylogenetic signal as expected from a BM model of evolutionary divergence (Fig. 4). In all cases, when the null‐model process and estimation matched (the Ω matrix was the same in the null‐model process and estimation), a 1:1 relationship between *P*‐values from parametric ANOVA and the nonparametric alternatives was evident. However, if either simulation or RRPP was used as a null‐model process along with GLS and λ=1, the correlation was not as strong for data simulated with λ≠1, as it was if λ was optimized. It was also quite apparent that if simulation was used as a null‐model process, it was important to rescale C in order to retain a linear relationship between *P*‐values (from parametric *F*‐statistics).

There were no obvious relationships among different estimation methods unless incidentally because data were simulated to have no phylogenetic signal (λ=0) or phylogenetic signal expected with a BM model of evolutionary divergence (λ=1), in which case the *P*‐values from GLS with optimized λ and either OLS, or GLS with λ=1, respectively, were highly correlated. These results only reinforce that λ optimization is an important step that yields consistent results with OLS and traditional PGLS estimation, at the extremes. Additionally, using OLS estimation when data had phylogenetic signal appeared to produce *P*‐values that were consistently near 0, regardless of null‐model process.

These patterns were generally the same among the nonparametric tests for effect sizes (*Z*‐scores; see Supporting Information); though here there is no basis for comparison to parametric tests, which do not have an obvious transformation to obtain a *Z*‐score. Collectively, these results confirm that the choice of null‐model process is not as important as estimation, provided λ optimization is performed at all stages. Ignoring optimization, under no circumstances does simulation from a BM model of evolutionary divergence as a null‐model process coupled with OLS estimation make sense. Rather, estimation and null‐model process should be seen as an analytical pairing that requires matching C matrices (inherent in RRPP), which performs best if C is scaled by λ.

**Figure 5:**
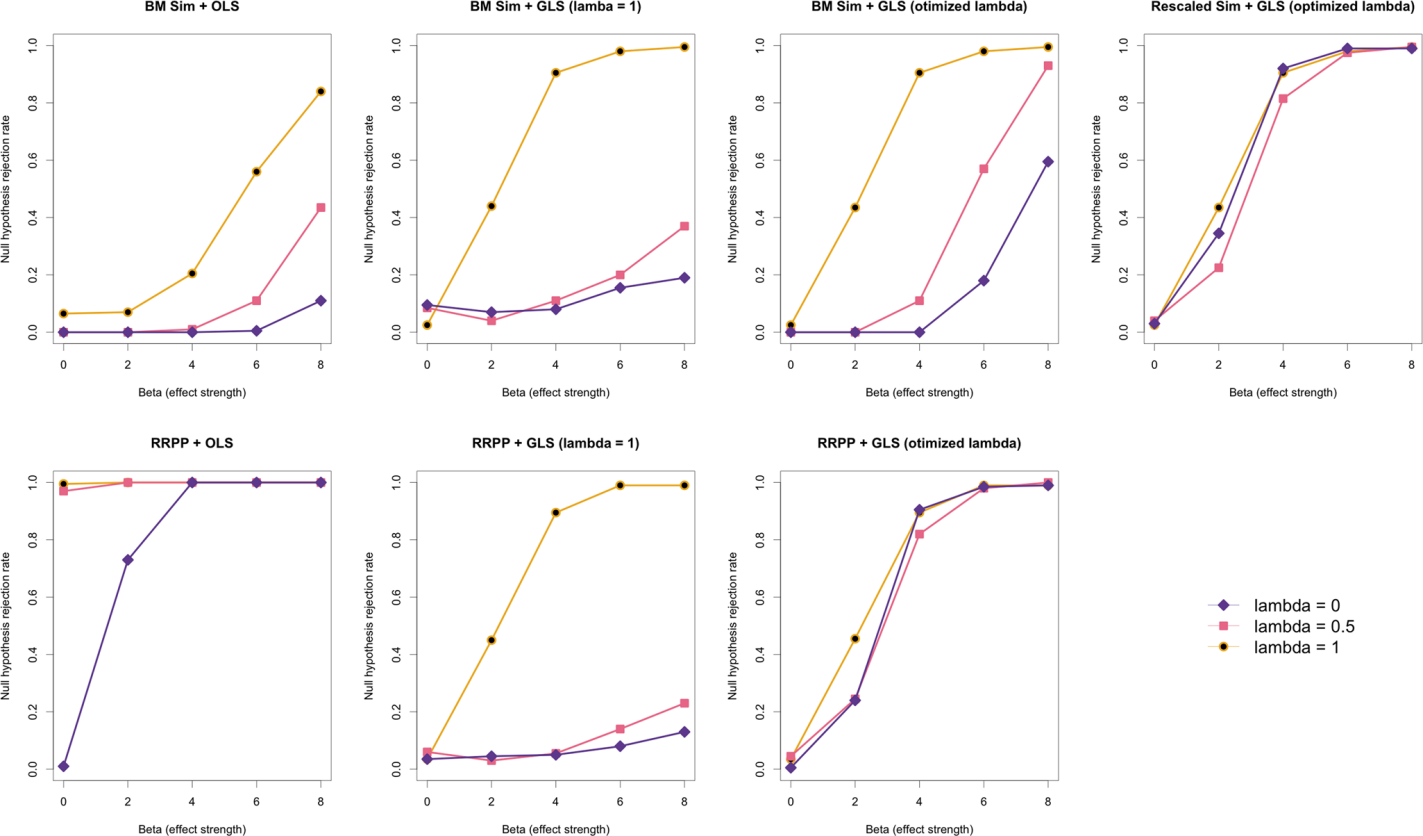
Statistical power curves for all combinations of estimation and null‐model process, and for the three data types based on λ. Null hypothesis rejection rates (each point) were based on 200 simulations.

*Comparison of statistical power*. Some of the inconsistencies noted in the first set of simulations were more obvious as pathologies in the second set of simulations, meant to address type I error and statistical power. Estimation with OLS or GLS without λ optimization tended to result in higher type I error rates (Fig. 5), unless the λ assumed for estimation happened to match the λ used to simulate data. For example, data simulated with λ=0 had appropriate type I error rates (at β=0) for OLS estimation and RRPP, but OLS estimation used on data with any phylogenetic signal had exceptionally large type I error rates; data simulated to have phylogenetic signal consistent with a BM model of evolutionary divergence had appropriate type I error rates if GLS was performed with λ=1, but had elevated type I error rates for data simulated with λ<1, with the rate increased for data simulated with λ=0 compared to λ=0.5 (further departure from λ=1). The only obvious difference between null‐model processes was that OLS estimation with RRPP (intrinsic relationship between residuals and estimation) and OLS estimation with BM data simulation had strikingly different results. Type I error rates were appropriate, irrespective of data type, if simulation was used. If simulation and RRPP assumed the same C matrix, type I error rate and power curves were indistinguishable (Fig. 5).

For statistical power, the method of estimation appeared to be more important than the null‐model process. This can be appreciated by the consistency of statistical power curves among the different data types, irrespective of null‐model process, juxtaposed with the disparity among power curves if GLS was not based on optimization of λ. Regarding the latter, statistical power was considerably higher if the value of λ used for GLS estimation incidentally matched the λ used to simulate data. A greater departure from this (λ=0) resulted in a greater reduction in statistical power. The simulated effect (β) also contributed to differences in statistical power. The only disparity between statistical power curves occurred at small β (2 or 4), with data simulated with stronger phylogenetic signal having greater statistical power.

The result of previous research (Adams and Collyer 2018b) was also confirmed; BM simulation as a null‐model process with OLS estimation (sim‐pANOVA) has less statistical power than RRPP with GLS, but only if data were simulated with a BM model of evolutionary divergence. The enhanced statistical power of RRPP with GLS appears to be an amelioration afforded by simulating only data with a BM model of evolutionary divergence. Simulating data with weaker phylogenetic signal renders low statistical power using GLS, assuming λ=1. However, optimizing λ provides the most statistical power, regardless of null‐model process or the apparent amount of phylogenetic correlation in the data. These results are consistent with those of previous work (Collyer et al. 2022), in which statistical power for detecting phylogenetic signal was increased by optimizing λ.

## Discussion

This research revealed several important points. First, whether simulation of data (residuals, more precisely) or RRPP is used as a null‐model process is inconsequential. Both methods produce reliable results. Second, the method of estimation is exceedingly important. Performing ANOVA with a method of estimation that does not appropriately estimate the phylogenetic covariances of the data can be detrimental. Statistical research varying the strength of phylogenetic signal has been performed for evaluating methods that test phylogenetic signal strength (e.g., Münkemüller et al. 2012; Collyer et al. 2022) but might be comparatively rarer for research that evaluates the proficiency of methods to test hypotheses with linear models. One exception is the work of Clavel and Morlon (2020) who varied λ as we did in this study, for the comparison of various multivariate methods. In their comparisons, two methods mimicked methods used in this study. They used BM simulation with OLS estimation of residual covariance matrices and RRPP with GLS estimation of residual covariance matrices, assuming λ=1. The former used MANOVA statistics and the latter used univariate‐like *F*‐statistics (Collyer et al. 2015) for test statistics, but for a single variable, these statistics would be comparable to the BM simulation plus OLS and RRPP plus GLS with λ=1 cases we considered here. Clavel and Morlon (2020) noted that using RRPP plus GLS with λ=1 resulted in drastically increased statistical power but also high type I error rates, when considered for only the first principal component if data were simulated with a BM model of evolutionary divergence, but statistical power was otherwise considerably lower than with multivariate likelihood or penalized likelihood statistics, which used $\hat{\Omega }$ instead of C. Our results are remarkably consistent with theirs for the univariate consideration in this study, and suggest that the inherent optimization step in their statistical approach is the important component of the cross‐validated statistics they introduced. The null‐model process they used was similar to RRPP, except that residuals were not transformed prior to permutation but were transformed after permutation, unlike the recommendation of Commenges (2003) to maintain approximate second‐moment exchangeability, and C was used rather than $\hat{\Omega }$ for transformation. The issues we found with BM simulation plus OLS were also consistent with the issues they found (limited statistical power). Not only did we find consilience of ANOVA methods for our comparisons of parametric ANOVA, simulation‐based ANOVA, and RRPP‐based ANOVA, when $\hat{\Omega }$ is based on an optimized value of λ, but we found some consistency with the increased statistical power of other multivariate methods that use $\hat{\Omega }$ rather than C.

Performing statistical research to evaluate methods that should have more applicability for multivariate data by simulating univariate data might seem unintuitive. However, there were two important reasons for doing this. First, simulating univariate data meant we could map random *F*‐statistics on the density plot of parametric *F*‐distributions to consider the behavior of the different combinations of null‐model process and estimation. We could have alternatively considered examples with conditions that multivariate statistics have exact *F*‐distributions but this would have also meant generalizing sim‐pANOVA—a commonly used method—for MANOVA. Second, and more importantly, generalizing the maximum‐likelihood estimation of λ for multivariate data is not a trivial exercise. As noted by Collyer et al. (2022), λ optimization can be quite complex, starting with the consideration of whether λ is free to vary across multivariate variables. Finding the determinant of a residual covariance matrix, |Σ|, is essential for estimating model likelihood. Σ is found for multivariate data as, $R⊗\hat{\Omega }$, where ⊗ indicates a Kronecker product, and R is the residual covariance matrix generalization of ${\hat{\sigma }}^{2}$, found from an n×p matrix of data, Y, as $R={\left(n-k\right)}^{-1}{\left(Y-X\hat{\beta }\right)}^{T}{\hat{\Omega }}^{-1}\left(Y-X\hat{\beta }\right)$. However, this definition implies single R and $\hat{\Omega }$ matrices, which means a scalar λ must be used in estimation. Allowing for *p* different R and $\hat{\Omega }$ matrices is possible with an algorithm to solve Σ for likelihood estimation, with maximum‐likelihood estimates of λ first found *p* times for each variable (Collyer et al. 2022), a likely computationally intense procedure.

Clavel and Morlon (2020) offered that a maximum‐likelihood estimate of Ω could be made with either a scalar or vector of λ, but it is not clear how either would be obtained. Whether λ should be a scalar or vector must consider the type of multivariate data (see Adams and Collyer 2018a, 2019). For example, shape data found through generalized Procrustes analysis (Rohlf and Slice 1990) uses multiple variables to describe one organism attribute, shape. Thus, assuming that natural selection acts independently on subcomponents of a set of shape variables—as is explicitly the case with optimizing separate λ for each trait dimension—would not make much sense. Thus, for such multidimensional traits, use of a common scalar of λ would be more appropriate. Yet this recommendation is in contrast to that for, say, life‐history data, where a multivariate dataset comprises multiple traits that could (in theory) be independent. Here, a separate λ for each trait may be envisioned (for a similar approach with evolutionary rates, see Adams 2013). Although a scalar λ offers a simpler calculation of Σ, and thus, a simpler estimation of model likelihood, allowing independent λ optimization offers a potentially simpler solution for optimization, as λ is optimized for each variable with a univariate optimization strategy. It is the multivariate generalization of a single λ parameter that is potentially more complex.

Although the optimization of λ is straightforward—the value of λ that maximizes the likelihood estimator—calculating the likelihood is fraught with estimation issues as Σ becomes singular (when the number of variables approaches or exceeds the number of observations). In this study, using univariate examples posed no issue, but generalizing the maximum‐likelihood estimate for λ for multivariate data would require further investigation, especially for high‐dimensional data, in which case Σ is certainly singular but tractable test statistics based on traces of residual covariance matrices could be used. Clavel and Morlon (2020) introduced penalized likelihood, which offers an ability to find $\hat{\lambda }$, even for high‐dimensional data, but this approach might only be useful for likelihood‐based statistics as test statistics.

We envision four possible scenarios for generalizing λ optimization to find a scalar that maximizes the likelihood of a model for high‐dimensional data (for the general formula for multivariate likelihood estimation, see Revell and Harmon 2008), and for test statistics that do not necessarily rely on calculating model likelihood. The simplest generalization—if data dimensionality is not an issue—finds alternative multivariate likelihoods based on residual covariance matrix estimates, spanning λ from 0 to 1. However, this approach could fail to consider strong latent phylogenetic signal, restricted to only a portion of the data space (see Collyer and Adams 2021a). For example, for shape data where strong phylogenetic signal is localized to a portion of a more comprehensive anatomical configuration, a solution that converges toward λ=0 might be found. (In this case, allowing λ to vary might be warranted.) The second approach, which would mitigate the potential issues of the first approach, is to find optimized values of λ variable by variable, and average them. This solution, however, would bias λ optimization toward 0.5 unless all variables either have or lack phylogenetic signal. Based on our statistical power results (Fig. 5), this might not be such a worrisome outcome, as the most egregious issues occurred for cases where λ used in analysis had a large departure from its optimized value. (A tendency toward an intermediate value would preclude larger disparity that could exist between two λ values.)

The third alternative would be to determine the data dimensions that have most phylogenetic signal and rotate the data space with respect to these dimensions, an analysis called phylogenetically aligned component analysis (Collyer and Adams 2021a). Multivariate optimization of λ could thus be confined to the dimensions where phylogenetic signal is present. This procedure would likely bias λ in a positive direction, which could yield traditional PGLS solutions (assuming λ=1) even if phylogenetic signal is constrained to a small portion of the variables. Finally, the likely best solution is also the most computationally exhaustive. RRPP or simulation could be performed for a reliable number of permutations, for example, λ=0,0.1,0.2,…,1, and a spline function could find the optimal λ that maximizes a *Z*‐score for characterizing phylogenetic signal, sensu Collyer et al. (2022). This approach has used sampling distributions of log‐likelihood statistics measuring phylogenetic signal to obtain, *Z*, which has been shown to have a linear association with λ. Thus, measuring likelihood was less important than its role in measuring the phylogenetic signal effect size. The same approach could be used on *Z*‐scores obtained from distributions of random univariate‐like *F*‐statistics based on traces of residual covariance matrices. Whether such an approach yields comparable or better statistical power than penalized likelihood approaches would also be a useful future research endeavor.

If λ should be considered free to vary, a multivariate generalization may not be needed, as *p* univariate solutions would be acquired for *p* variables. However, this approach also assumes that each variable could be considered independent, which is perhaps a risky assumption. An alternative solution is to use the independent solutions as a starting point for an iterative procedure that finds the optimal combination of values that maximizes likelihood, sensu Adams (2013). Such an approach might have intrigue as an analysis that considers the modularity of anatomical subconfigurations for landmark shape data (sensu Klingenberg 2009; Adams 2016; Zelditch and Goswami 2021), as suites of contrasting λ values for groups of variables might be evidence for natural selection acting differently on anatomical components.

This discussion highlights the current tension and needed research direction. Although research to evaluate the most appropriate methods to estimate Ω for multivariate data, especially with respect to statistics used for hypothesis tests, will require a thorough investigation, what can researchers do currently to assuage concerns about inappropriately estimated Ω matrices? As a heuristic, at least for a scalar form of λ, simply performing a range of analyses first with, for example, λ=0,0.1,0.2,…,1 for a linear model that contains only an intercept, and choosing a value of λ that yields the largest effect size, *Z*, based on a distribution of random log‐likelihoods (Collyer et al. 2022) will be a valuable analytical strategy. This is consistent with one of the proposed optimization strategies we outlined above. Furthermore, if it is found that alternative optimization strategies work well, the advantage would be in saved computation time but likely not improved statistical power, over this approach. It can be expected that a solution that maximizes *Z* also maximizes likelihood, using RRPP (Collyer et al. 2022). If one wishes to allow λ to vary among different variables, then finding λ variable by variable might not maximize model likelihood compared to an alternative solution, but might have a solution that maximizes model likelihood better than a scalar. As a minimum, these are two approaches that should work well—especially compared to assuming λ=1 in PGLS or using OLS in sim‐pANOVA—and could possibly be improved with further research.

Given that estimation is important, the question turns to whether residuals should be resampled or simulated, as a null process? We have shown there is no real analytical concern, as results will be consistent with simulated data, but under which conditions would this question be swayed to a particular answer? Simulation assumes a parametric distribution from which data are sampled. RRPP uses the residuals that are calculated. An advantage to simulation is it can be assured that the null process correctly asserts an appropriate distribution. An advantage to RRPP is that an assumption about the distribution of residuals is not required. It will require further research to evaluate if the two methods have contrasting results with residuals that are not normally distributed or are heteroscedastic. However, if the two methods can be relied on to produce consistent results, shuffling transformed residuals is probably computationally faster than simulating residuals from a null distribution that must be modified to have phylogenetic correlation in each permutation.

We feel that a few potential updates to software packages that offer either sim‐pANOVA or RRPP‐ANOVA should be strongly considered. First, estimation of Ω based on an optimized λ should be made available. If data are simulated, rescaling the phylogenetic tree used for simulation by $\hat{\lambda }$ should be an essential step. Simulation should also simulate residuals that are added to null‐model fitted values, which are estimated with PGLS, with $\hat{\Omega }$ based on $\hat{\lambda }$, rather than the simulation of new data in every permutation. This is especially true for linear models with multiple effects. Currently, software packages that offer sim‐pANOVA do so only for single‐factor models, in which case simulating new data is no different from simulating residuals. However, for multiple linear model effects, multiple null models and therefore, multiple null‐model processes are required. The simulation of new data implicitly considers the same model with only an estimated mean as a null model, which would probably not make much sense for ANOVA based on type I, type II, or type III sums of squares and cross‐products.

More broadly, our work exposed the fact that when evaluating macroevolutionary trends across a phylogeny, it is the appropriate conditioning of the data on the phylogeny during the analysis, and not phylogenetic simulation alone, which was responsible for obtaining adequate sampling distributions (which should be a goal for making correct biological inferences). That is, the use of OLS estimation—as is common in some implementations of sim‐pANOVA—yields incorrect sampling distributions, and could thus lead to incorrect statistical inferences regarding patterns in cross‐species data. Although previous research has illustrated that GLS estimation is an obvious improvement, we have shown that PGLS assuming λ=1 can also be fraught with type I error rate and statistical power issues. Realizing that $\hat{\lambda }=0$ and $\hat{\lambda }=1$ are possible optimization outcomes, PGLS using $\hat{\lambda }$ should be viewed as a universal solution. Our research emphasized that statistical inference via PCMs requires statistical methods that condition data on the phylogeny, not merely data that are simulated from a phylogenetic process alone. From this it follows that simulation‐based approaches to macroevolutionary inference must account for phylogenetic nonindependence at two stages: the generation of random samples via simulation (e.g., Martins and Garland 1991; Garland et al. 1993), and in the analytics that are used to obtain statistics (e.g., Martins and Hansen 1997). When both of these conditions are met, macroevolutionary inferences derived from simulation‐based approaches are appropriate and reliable.

## CONFLICT OF INTEREST

The authors declare that there is no conflict of interest.

## AUTHOR CONTRIBUTIONS

Dean C. Adams and Michael L. Collyer collaboratively developed the concept and contributed to all portions of this manuscript. All authors approve of the final product and are willingly accountable for any portion of the content.

Associate Editor: T. Stayton

Handling Editor: M.L. Zelditch

## Supporting information

Figure S1: Density plots for random F‐statistics, from different combinations of estimation and null model process.
Figure S2: Pairs plots for P‐values from different combinations of estimation and null model process, for data generated with lambda = 0.
Figure S3: Pairs plots for P‐values from different combinations of estimation and null model process, for data generated with lambda = 0.5.
Figure S4: Pairs plots for P‐values from different combinations of estimation and null model process, for data generated with lambda = 1.
Figure S5: Statistical power curves for all combinations of estimation and null model process, and for the three data types based on lambda. Null hypothesis rejection rates (each point) were based on 200 simulations.

## Data Availability

R‐scripts for simulation tests are available in the Supplemental Information.
