# Supplementary material for: Consilience of methods for phylogenetic analysis of variance
Source: Evolution. 2022 May 19;76(7):1406–19. doi: 10.1111/evo.14512 (PMC9544334; doi:10.1111/evo.14512)
Supplement: Supplementary file 1 — Figure S1: Density plots for random F‐statistics, from different combinations of estimation and null model process. Figure S2: Pairs plots for P‐values from different combinations of estimation and null model process, for data generated with lambda = 0. Figure S3: Pairs plots for P‐values from different combinations of estimation and null model process, for data generated with lambda = 0.5. Figure S4: Pairs plots for P‐values from different combinations of estimation and null model process, for data generated with lambda = 1. Figure S5: Statistical power curves for all combinations of estimation and null model process, and for the three data types based on lambda. Null hypothesis rejection rates (each point) were based on 200 simulations. [file EVO-76-1406-s001.pdf]

Consilience of methods for phylogenetic analysis of variance: Supporting information

Dean C. Adams and Michael L. Collyer

This supporting information simultaneously provides code for analyses carried out in the main article and provides additional figures. All source functions can be found in the R-script, `Adams&Collyer.2022.source.R`.

```
source("Adams&Collyer.2022.source.R")
```

The simulations and analyses performed for the main article can require a lot of time. Computation time can be drastically decreased with parallel processing. The following code is designed to use parallel processing for Unix systems, via forking. A similar approach could be made on Windows systems, but requires making socket clusters. We found for the parameters used in the main article, parallel processing with a socket cluster approach offered no time savings. An update to use a socket cluster approach would require also updating the functions in `Adams&Collyer.2022.source.R`.

```
library(parallel)
spare_cores <- 2 # Number of cores to spare during processing
no_cores <- detectCores() - spare_cores
Unix <- .Platform$OS.type == "unix" # Check if OS is Unix

# Discern between Windows (forced no_cores <- 1)
# and Unix for parallel processing mode
PAR <- if(Unix) no_cores else 1
```

Comparison of sampling distributions.

The following parameters were used for analyses presented in the article, comparing sampling distributions:

```
set.seed(1969)

ng <- 25
g <- 10
x <- factor(rep(1:g, each = ng))
X <- model.matrix(~x + 0)

p <- 1

iter <- 99
nsims <- 100
```

```
beta <- 0
Beta <- matrix(0, ncol(X), p)
Beta[1,] <- beta
```

100 random trees were simulated:

```
trees <- lapply(1:nsims, function(.) pbtree(n = ng * g))
```

Data were simulated for $\lambda = 0, 0.5, 1$, for every simulation run. A step was used to make sure that simulated λ was within 2% of intended λ:

```
Data0 <- getData(nsims, lambda = 0, Beta, trees, PAR)
Data0.5 <- getData(nsims, lambda = 0.5, Beta, trees, PAR)
Data1 <- getData(nsims, lambda = 1, Beta, trees, PAR)
```

F-distributions were obtained for each combination of estimation method and null model process, for every simulation run, for the three data types:

```
Result0 <- getResult(Data0, PAR, iter)
Result0.5 <- getResult(Data0.5, PAR, iter)
Result1 <- getResult(Data1, PAR, iter)
```

The following creates density plots of random F-statistics and maps them on parametric F-distributions. This produces the same figure as Fig. 1 in the main article:

```
densityPlotMultiSims(Result0, Result0.5, Result1,
                     Cols = hcl.colors(3, palette = "Sunset",
                                       alpha = 0.1, rev = F),
                     par.Col = 1, par.Lwd = 1)

plot(c(0,1), c(0,1), type = "n", bty = "n",
     xlab = "", ylab = "", xaxt = "n", yaxt = "n")

legend("center", c("lambda = 0", "lambda = 0.5",
                   "lambda = 1", "Parametric F"), lty = 1, lwd = c(3,3,3,1),
       col = c(hcl.colors(3, palette = "Sunset",
                    alpha = 0.4, rev = F), 8), bty = "n", cex = 0.7)
```

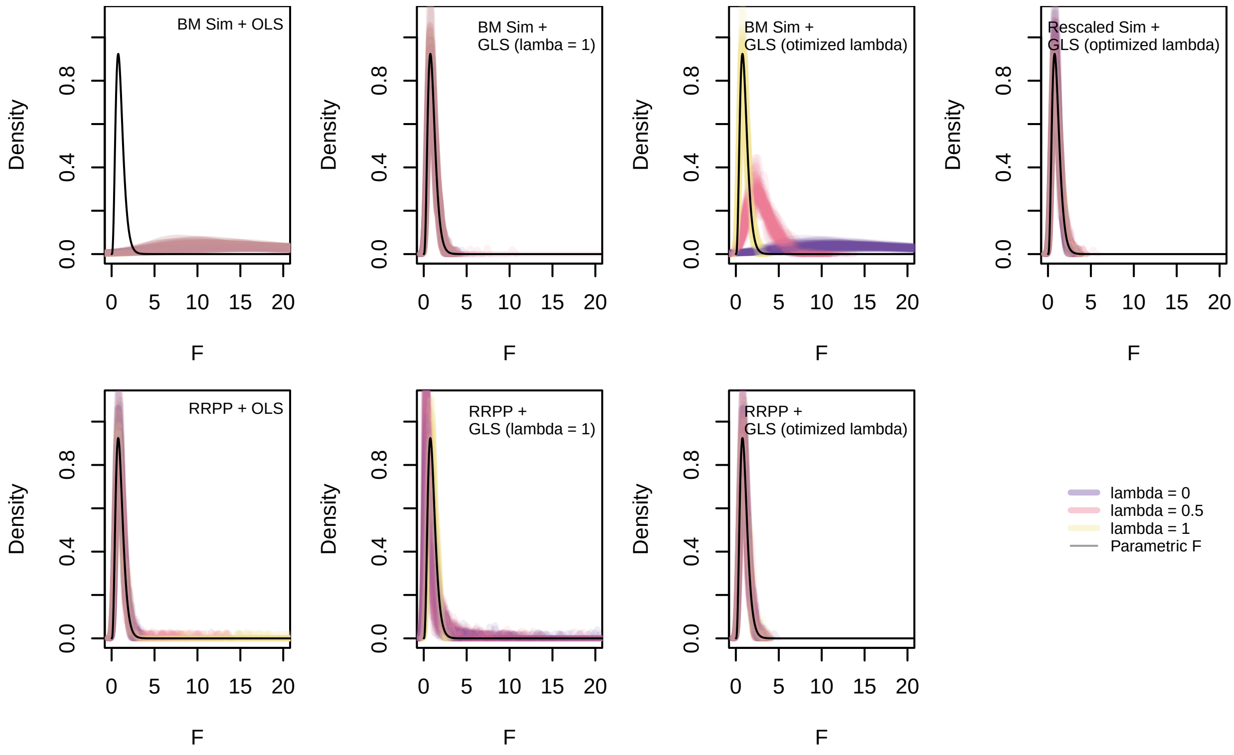

Figure S1: Density plots for random F-statistics, from different combinations of estimation and null model process. 100 density curves for every level of simulated lambda (colored differently) is overlayed in every frame. Parametric F-distributions are shown as black curves.

The following code acquires ANOVA statistics across simulation runs and produces "pairs" plots for comparison of P-values. These plots are the same as those in Figs. 2-4 of the main article, but they are sorted by null model process rather than method of estimation.

```
pairs.names <- c("Parametric \nOLS",
                 "Parametric \nGLS-BM",
                 "Parametric \nGLS-optimized",
                 "RRPP \nwith \nOLS",
                 "RRPP \nwith \nGLS-BM",
                 "RRPP \nwith \nGLS-optimized",
                 "BM Sim \nand \nOLS ",
                 "BM Sim \nand \nGLS-BM",
                 "BM Sim \nand GLS-\noptimized",
                 "Rescaled Sim \nand GLS-\noptimized")

Stats0 <- getStatsSims(Result0)
Stats0.5 <- getStatsSims(Result0.5)
Stats1 <- getStatsSims(Result1)

Cols = hcl.colors(3, palette = "Sunset",
                  alpha = 0.8, rev = F)

pd <- (cbind(Stats0$Ppar[, 1:3], Stats0$Prand))
colnames(pd) <- pairs.names
```

```
pairs(pd, pch = 19, cex = 0.7,
      cex.labels = 0.5, col = Cols[1],
      xlim = c(0, 1), ylim = c(0,1), upper.panel = NULL)
```

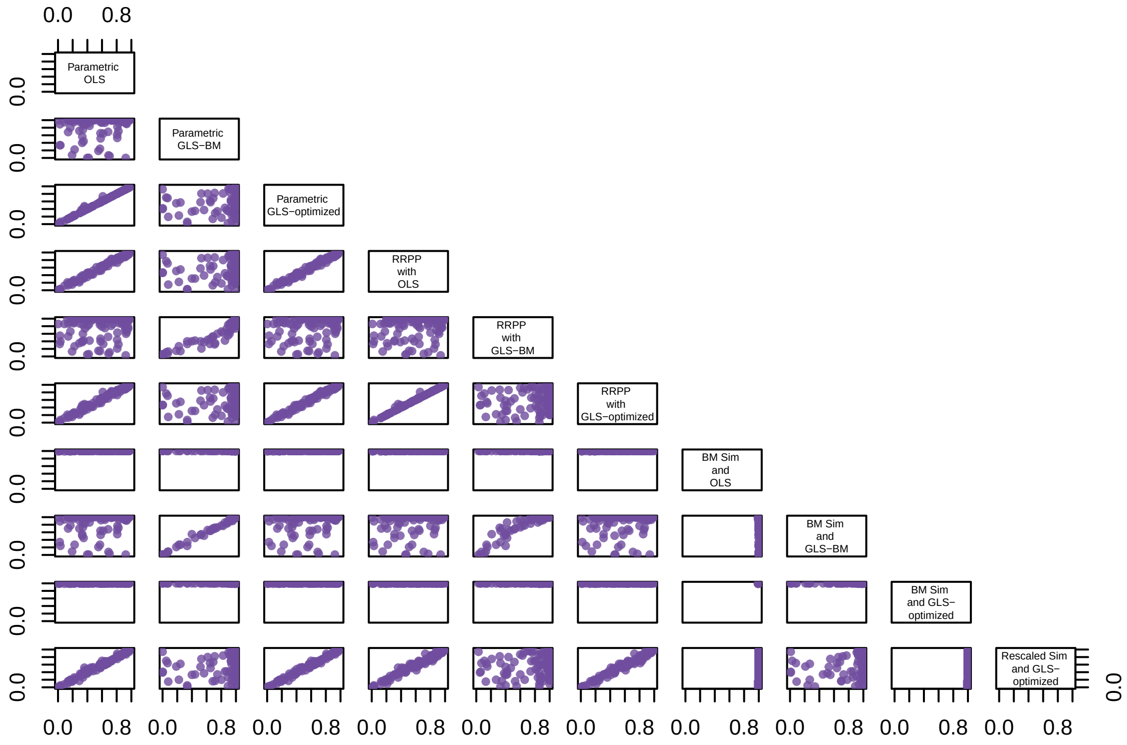

Figure S2: Pairs plots for P-values from different combinations of estimation and null model process, for data generated with lambda = 0.

```
pd <- (cbind(Stats0.5$Ppar[, 1:3], Stats0.5$Prand))
colnames(pd) <- pairs.names

pairs(pd, pch = 19, cex = 0.7,
      cex.labels = 0.5, col = Cols[2],
      xlim = c(0, 1), ylim = c(0,1), upper.panel = NULL)
```

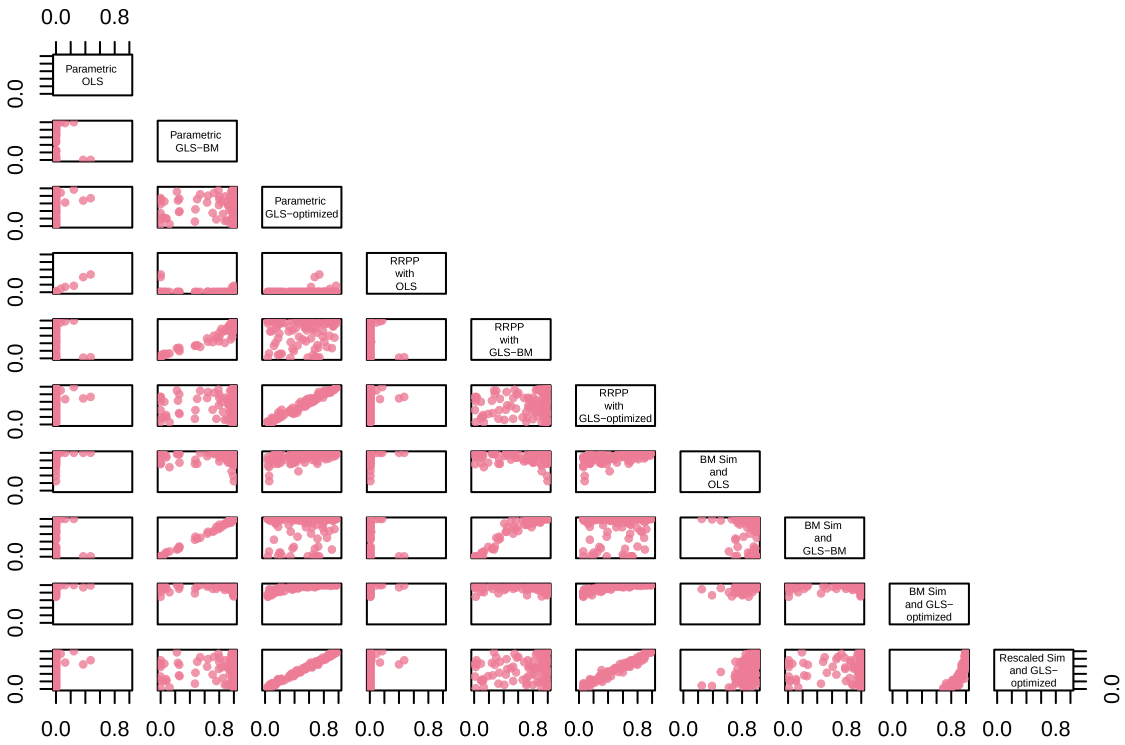

Figure S3: Pairs plots for P-values from different combinations of estimation and null model process, for data generated with lambda = 0.5.

```
pd <- (cbind(Stats1$Ppar[, 1:3], Stats1$Prand))
colnames(pd) <- pairs.names

pairs(pd, pch = 19, cex = 0.7,
      cex.labels = 0.5, col = Cols[3],
      xlim = c(0, 1), ylim = c(0,1), upper.panel = NULL)
```

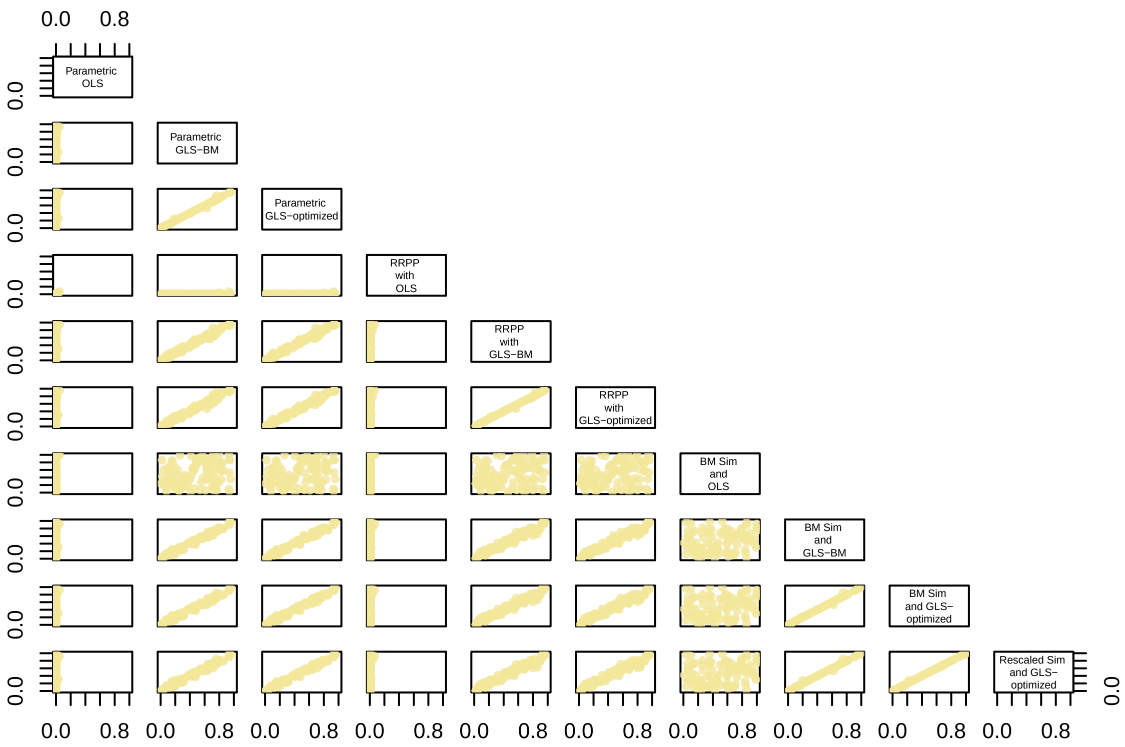

Figure S4: Pairs plots for P-values from different combinations of estimation and null model process, for data generated with lambda = 1.

The following code repeats the pairs plots, but this time for Z-scores. The parametric cases are excluded, as Z-scores require an empirical sampling distribution from which to calculate a standard deviation.

```
pd <- Stats0$Z
colnames(pd) <- pairs.names[-(1:3)]

pairs(pd, pch = 19, cex = 0.7,
      cex.labels = 0.5, col = Cols[1],
       upper.panel = NULL)
```

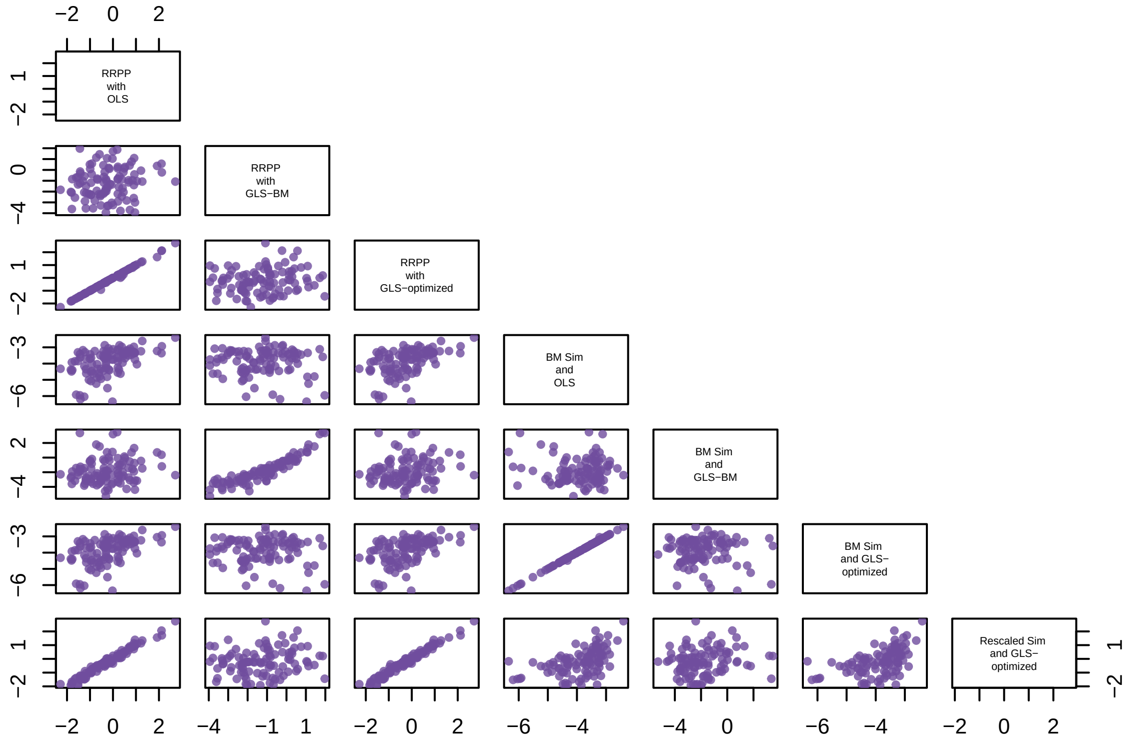

```
pd <- Stats0.5$Z
colnames(pd) <- pairs.names[-(1:3)]

pairs(pd, pch = 19, cex = 0.7,
      cex.labels = 0.5, col = Cols[2],
       upper.panel = NULL)
```

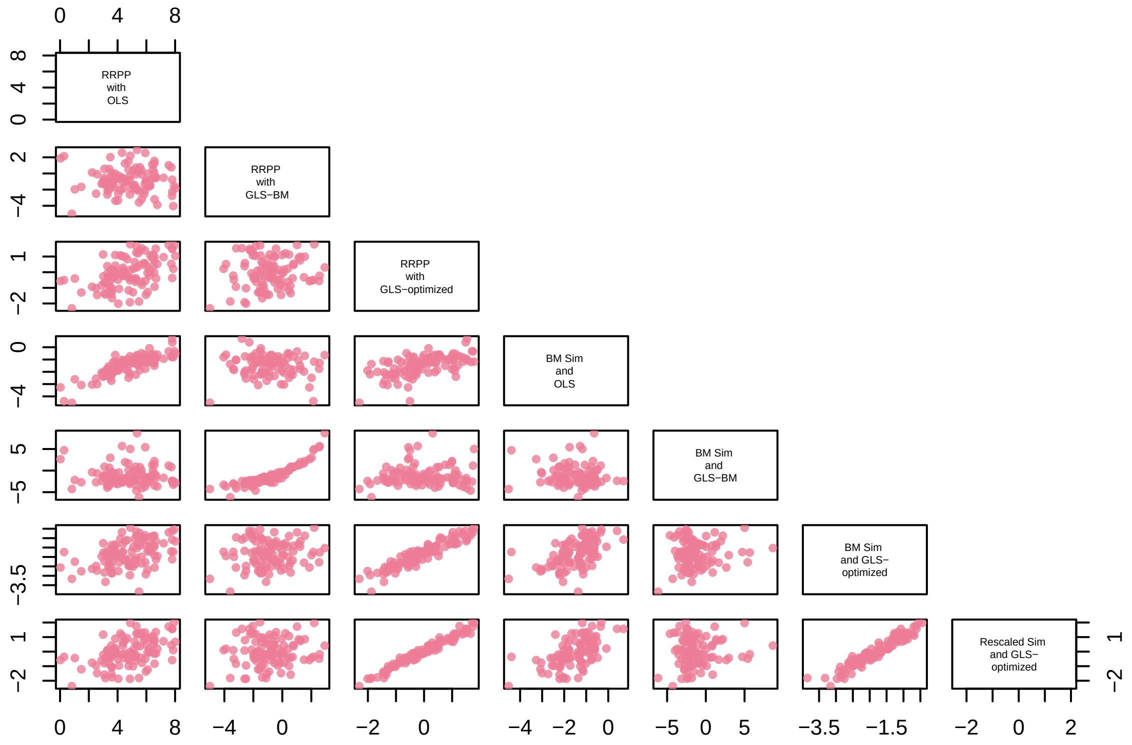

```
pd <- Stats1$Z
colnames(pd) <- pairs.names[-(1:3)]

pairs(pd, pch = 19, cex = 0.7,
      cex.labels = 0.5, col = Cols[3],
      upper.panel = NULL)
```

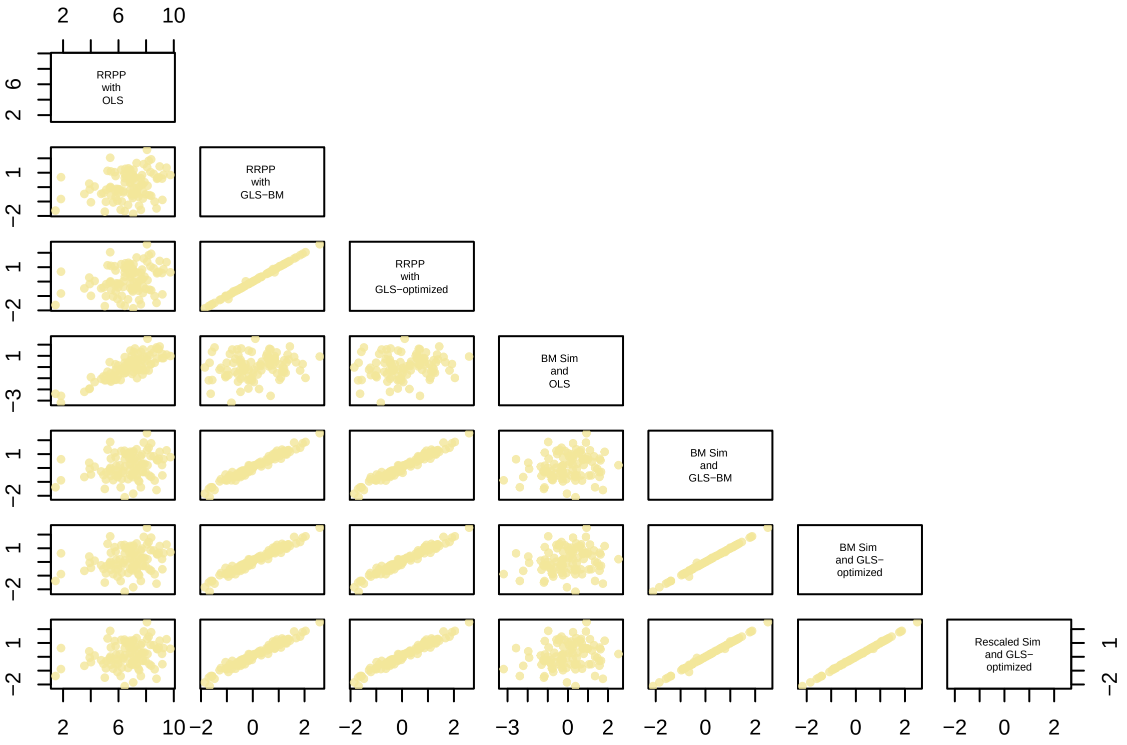

Comparison of statistical power.

The following parameters were used for analyses presented in the article, comparing type I error rates and statistical power:

```
set.seed(1969)

ng <- 25
g <- 10
x <- factor(rep(1:g, each = ng))
X <- model.matrix(~x + 0)

p <- 1

iter <- 99
nsims <- 200

betas <- seq(0, 8, 2)
Beta <- matrix(0, ncol(X), p)
```

200 random trees were simulated:

```
trees <- lapply(1:nsims, function(.) pbtree(n = ng * g))
```

Data were simulated for $\lambda = 0, 0.5, 1$, for every simulation run. A step was used to make sure that simulated λ was within 2% of intended λ:

```
Data0 <- lapply(1:length(betas), function(j){
  beta <- betas[[j]]
  Beta[1] <- beta
  getData(nsims, lambda = 0, Beta, trees, PAR)
})

Data0.5 <- lapply(1:length(betas), function(j){
  beta <- betas[[j]]
  Beta[1] <- beta
  getData(nsims, lambda = 0.5, Beta, trees, PAR)
})

Data1 <- lapply(1:length(betas), function(j){
  beta <- betas[[j]]
  Beta[1] <- beta
  getData(nsims, lambda = 1, Beta, trees, PAR)
})

names(Data0) <- names(Data0.5) <- names(Data1)
```

The following are frequency tables, counting the number of significant outcomes (null hypothesis rejections) at a significance level of $\alpha = 0.05$.

```
Result0 <- lapply(Data0, function(x)
  getResult(x, PAR, iter))
Result0.5 <- lapply(Data0.5, function(x)
  getResult(x, PAR, iter))
Result1 <- lapply(Data1, function(x)
  getResult(x, PAR, iter))

sigResults0 <- lapply(Result0,
                      function(x) getSigResults(x, alpha = 0.05))
sigResults0.5 <- lapply(Result0.5,
                      function(x) getSigResults(x, alpha = 0.05))
sigResults1 <- lapply(Result1,
                      function(x) getSigResults(x, alpha = 0.05))
```

Convert the tables from the previous step into rates.

```
type1.0 <- sapply(sigResults0, function(x)
  Reduce("+", x) / length(x))
type1.0.5 <- sapply(sigResults0.5, function(x)
  Reduce("+", x) / length(x))
type1.1 <- sapply(sigResults1, function(x)
  Reduce("+", x) / length(x))

colnames(type1.0) <- colnames(type1.0.5) <-
  colnames(type1.1) <- betas
```

Plot the results (same as Fig. 5 in main article).

```
par(mfrow = c(2, 4))

Cols = hcl.colors(3, palette = "Sunset",
                  alpha = 1, rev = F)
Cols[3] <- "goldenrod2"

mains <- c("BM Sim + OLS", "BM Sim + GLS (lamba = 1)",
           "BM Sim + GLS (otimized lambda)",
           "Rescaled Sim + GLS (optimized lambda)",
           "RRPP + OLS", "RRPP + GLS (lambda = 1)",
           "RRPP + GLS (otimized lambda)")


plot(betas, betas, ylim = c(0,1), xlab = "Beta (effect strength)",
     ylab = "Null hypothesis rejection rate",
```

```
     type = "n", main = mains[1], cex.main = 0.6)

points(betas, type1.1[4,], type = "o", pch = 21,
       col = Cols[3], bg = 1, cex = 1.5)
points(betas, type1.0.5[4,], type = "o", pch = 22,
       col = Cols[2], bg = Cols[2], cex = 1.5)
points(betas, type1.0[4,], type = "o", pch = 23,
       col = Cols[1], bg = Cols[1], cex = 1.5)

plot(betas, betas, ylim = c(0,1), xlab = "Beta (effect strength)",
     ylab = "Null hypothesis rejection rate",
     type = "n", main = mains[2], cex.main = 0.6)

points(betas, type1.1[5,], type = "o", pch = 21,
       col = Cols[3], bg = 1, cex = 1.5)
points(betas, type1.0.5[5,], type = "o", pch = 22,
       col = Cols[2], bg = Cols[2], cex = 1.5)
points(betas, type1.0[5,], type = "o", pch = 23,
       col = Cols[1], bg = Cols[1], cex = 1.5)

plot(betas, betas, ylim = c(0,1), xlab = "Beta (effect strength)",
     ylab = "Null hypothesis rejection rate",
     type = "n", main = mains[3], cex.main = 0.6)

points(betas, type1.1[6,], type = "o", pch = 21,
       col = Cols[3], bg = 1, cex = 1.5)
points(betas, type1.0.5[6,], type = "o", pch = 22,
       col = Cols[2], bg = Cols[2], cex = 1.5)
points(betas, type1.0[6,], type = "o", pch = 23,
       col = Cols[1], bg = Cols[1], cex = 1.5)

plot(betas, betas, ylim = c(0,1), xlab = "Beta (effect strength)",
     ylab = "Null hypothesis rejection rate",
     type = "n", main = mains[4], cex.main = 0.6)

points(betas, type1.1[7,], type = "o", pch = 21,
       col = Cols[3], bg = 1, cex = 1.5)
points(betas, type1.0.5[7,], type = "o", pch = 22,
       col = Cols[2], bg = Cols[2], cex = 1.5)
points(betas, type1.0[7,], type = "o", pch = 23,
       col = Cols[1], bg = Cols[1], cex = 1.5)

plot(betas, betas, ylim = c(0,1), xlab = "Beta (effect strength)",
     ylab = "Null hypothesis rejection rate",
     type = "n", main = mains[5], cex.main = 0.6)

points(betas, type1.1[1,], type = "o", pch = 21,
       col = Cols[3], bg = 1, cex = 1.5)
points(betas, type1.0.5[1,], type = "o", pch = 22,
       col = Cols[2], bg = Cols[2], cex = 1.5)
points(betas, type1.0[1,], type = "o", pch = 23,
       col = Cols[1], bg = Cols[1], cex = 1.5)
```

```
plot(betas, betas, ylim = c(0,1), xlab = "Beta (effect strength)",
     ylab = "Null hypothesis rejection rate",
     type = "n", main = mains[6], cex.main = 0.6)

points(betas, type1.1[2,], type = "o", pch = 21,
       col = Cols[3], bg = 1, cex = 1.5)
points(betas, type1.0.5[2,], type = "o", pch = 22,
       col = Cols[2], bg = Cols[2], cex = 1.5)
points(betas, type1.0[2,], type = "o", pch = 23,
       col = Cols[1], bg = Cols[1], cex = 1.5)

plot(betas, betas, ylim = c(0,1), xlab = "Beta (effect strength)",
     ylab = "Null hypothesis rejection rate",
     type = "n", main = mains[7], cex.main = 0.6)

points(betas, type1.1[3,], type = "o", pch = 21,
       col = Cols[3], bg = 1, cex = 1.5)
points(betas, type1.0.5[3,], type = "o", pch = 22,
       col = Cols[2], bg = Cols[2], cex = 1.5)
points(betas, type1.0[3,], type = "o", pch = 23,
       col = Cols[1], bg = Cols[1], cex = 1.5)

plot(betas, betas, type = "n", bty = "n", xaxt = "n",
     yaxt = "n", xlab = "", ylab = "")

legend("center", c("lambda = 0", "lambda = 0.5", "lambda = 1"),
       lty = 1, col = Cols, pch = c(23,22,21),
       pt.bg = c(Cols[1:2], 1), bty = "n", cex = 0.7)
```

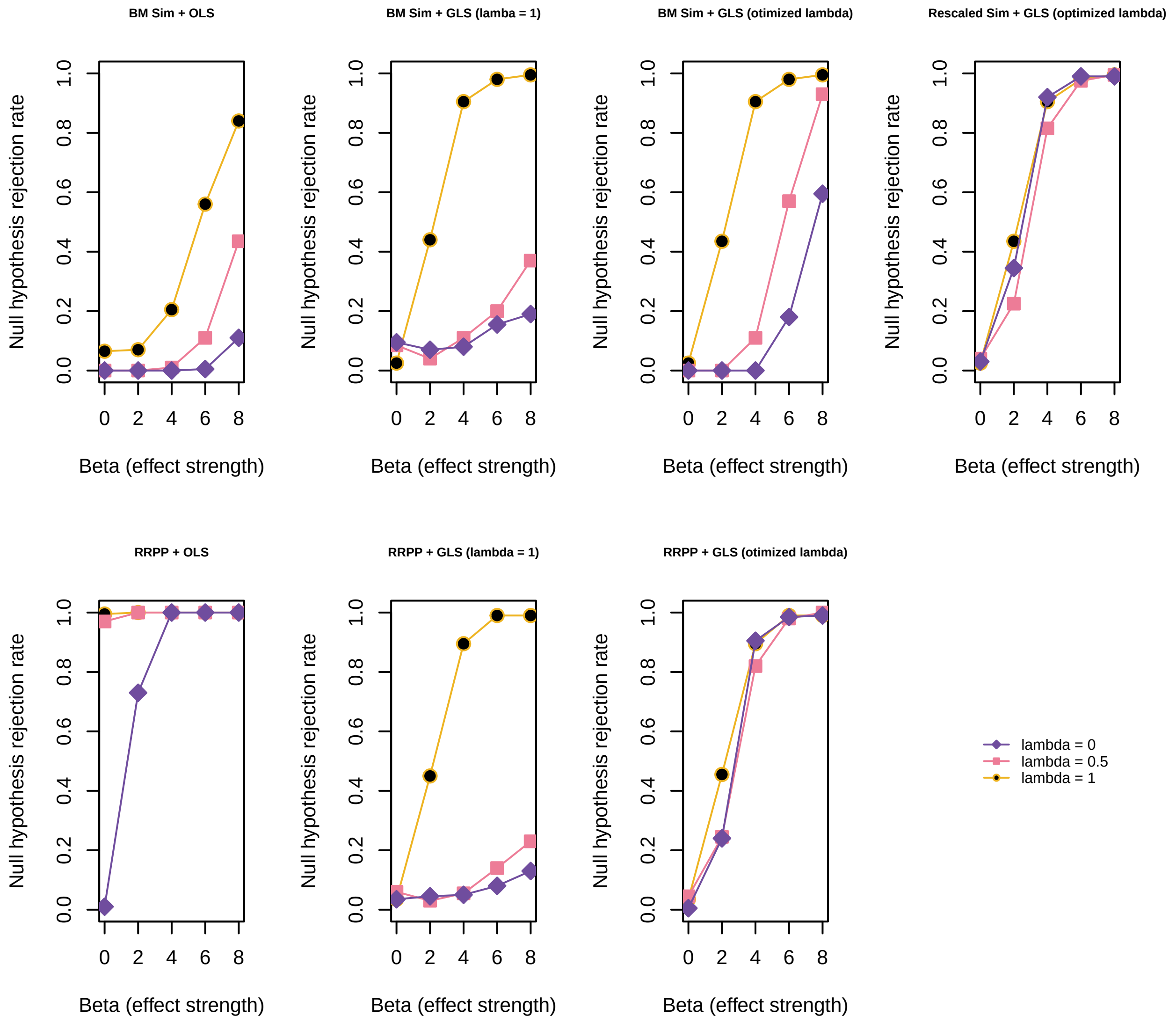

Figure S5: Statistical power curves for all combinations of estimation and null model process, and for the three data types based on lambda. Null hypothesis rejection rates (each point) were based on 200 simulations.
